# Gout gone awry: The importance of proper diagnosis

**DOI:** 10.1002/ccr3.5201

**Published:** 2021-12-16

**Authors:** Sophia Tessema, Abdullahi E. Mahgoub, Rasha Nakhleh

**Affiliations:** ^1^ Hurley Medical Center Neuro‐Ophthalmology Fellow, Michigan State University East Lansing MI USA; ^2^ Hurley Medical Center Geriatric Fellow, Geriatric Medicine Department Flint MI USA; ^3^ Division of General Internal Medicine & Geriatrics Oregon Health & Science University Portland OR USA

**Keywords:** geriatric medicine, health maintenance, orthopedics, pharmacology

## Abstract

Gout is the best‐known type of arthritis with a prevalence of 1%–3% in the western world (Therapeutic Advances in Musculoskeletal Disease, 6, 2014 and 131; Journal of Advanced Research, 8, 2017 and 495). Although it is well understood, there is growing evidence of the misdiagnosis of gout from other forms of arthritis. These errors lead to delay in accurate diagnosis (Journal of Advanced Research, 8, 2017 and 495).

## Graphical abstract

Gout is the best‐known type of arthritis with a prevalence of 1%–3% in the western world (Therapeutic Advances in Musculoskeletal Disease, 6, 2014 and 131; Journal of Advanced Research, 8, 2017 and 495). Although it is well understood, there is growing evidence of the misdiagnosis of gout from other forms of arthritis. These errors lead to delay in accurate diagnosis and in appropriate care for patients with gout (Journal of Advanced Research, 8, 2017 and 495).

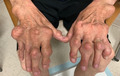

## 1 CASE DESCRIPTION

A 76‐year‐old Caucasian man presented to the clinic regarding gait abnormalities for the last 4 months, pain in the arch of his feet, numbness, and discomfort in multiple joints. The patient noted pain and stiffness in his wrists 17 years ago. He was evaluated and diagnosed with osteoarthritis. Since that time, he managed his pain with meloxicam. Twelve years later, he noticed the sudden onset of nodules in his joints, reduced range of motion, and stiffness impairing his gait. The patient had no prior investigation of gout and believed his symptoms were due to osteoarthritis.

**FIGURE 1:**
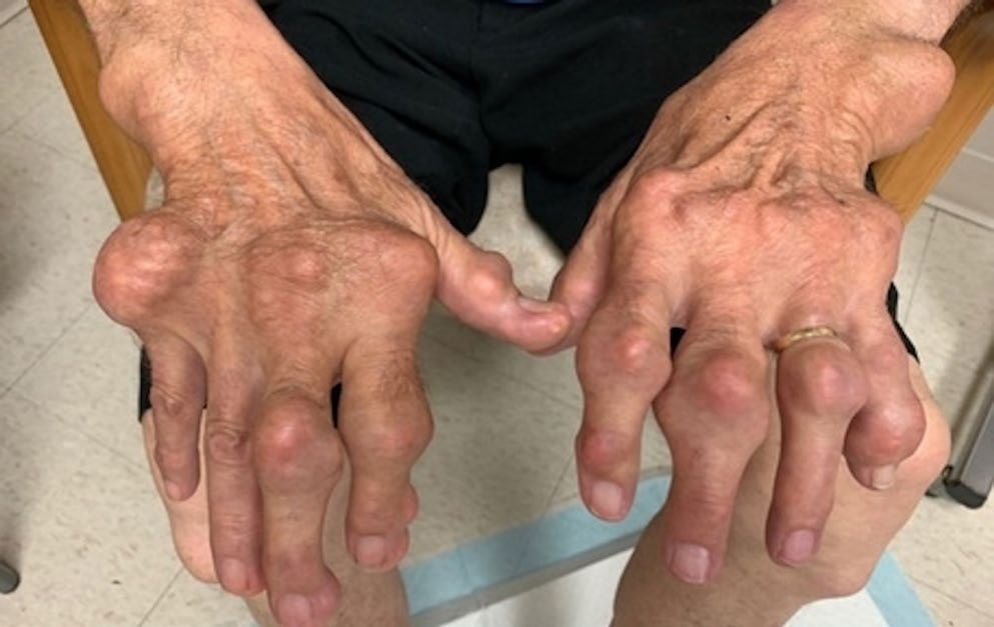
Diffuse and prominent soft‐tissue tophi

**FIGURE 2:**
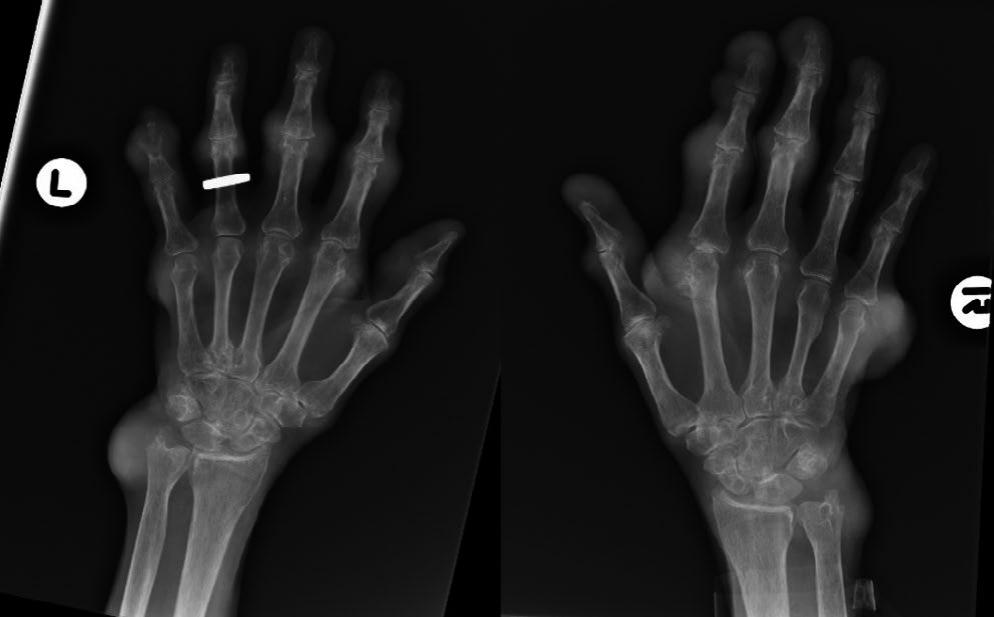
Destructive changes involving distal half of the intermediate phalanx of the left fifth finger in the proximal half of the distal phalanx. Prominent soft‐tissue swelling at the PIP joints of the index, middle, and ring fingers. Marked soft‐tissue prominence medially to the distal aspect of the ulna and erosive changes of the ulnar styloid. Marked soft‐tissue prominence present at right DIP joint of thumb, index finger, middle finger, PIP joints of the index, and middle finger. Faint erosive changes in index finger MP joint. Second and fifth MP joints demonstrate marked soft‐tissue prominence

The physical examination revealed bilateral large firm tophi on his hands, elbows, knees, and feet with ulceration and chalky white discharge noted on his index fingers (Figure 1, Figure S1, Figure S2, Figure S3, Figure S4a, and Figure S4b). Radiologic X‐ray films noted diffuse soft‐tissue tophi, destructive, as well as diffuse tophi of the feet (Figure 2, Figure S5). Laboratory workup revealed a slightly increased uric acid level of 8.6 mg/dl. Gout is the best known type of arthritis with a prevalence of 1‐3% in the western world.^1^, ^2^ There is growing evidence of misdiagnosis which leads to delay in accurate diagnosis and appropriate treatment.^2^The patient was started on Allopurinol and later switched to Pegloticase infusion therapy. Significant improvement of joint stiffness and tophus size was noticed 6 months after the continuation of therapy.

## CONFLICT OF INTEREST

The authors have declared that no conflict of interest exists.

## AUTHOR CONTRIBUTIONS

All the authors made substantial contributions to the preparation of this manuscript and approved the final version for submission.

## CONSENT

Informed consent has been obtained for the publication of this clinical image. Written informed consent was obtained from the patient to publish this report in accordance with the journal’s patient consent policy.

## Supporting information

Figure S1

Figure S2

Figure S3

Figure S4A

Figure S4B

Figure S5

Figure S6

## Data Availability

Data sharing not applicable – no new data generated, or the article describes entirely theoretical research.

## References

[1] Chowalloor PV , Siew TK , Keen HI . Imaging in gout: a review of the recent developments. Ther Adv Musculoskelet Dis. 2014;6(4):131–143. doi:10.1177/1759720X14542960 25342993 PMC4206657

[2] Ragab G , Elshahaly M , Bardin T . Gout: an old disease in new perspective – A review. J Adv Res. 2017;8(5):495–511. doi:10.1016/j.jare.2017.04.008 28748116 PMC5512152

