# Supplementary figures and images for: Gout gone awry: The importance of proper diagnosis
Source: Clin Case Rep. 2021 Dec 16;9(12):e05201. doi: 10.1002/ccr3.5201 (PMC8677883; doi:10.1002/ccr3.5201)

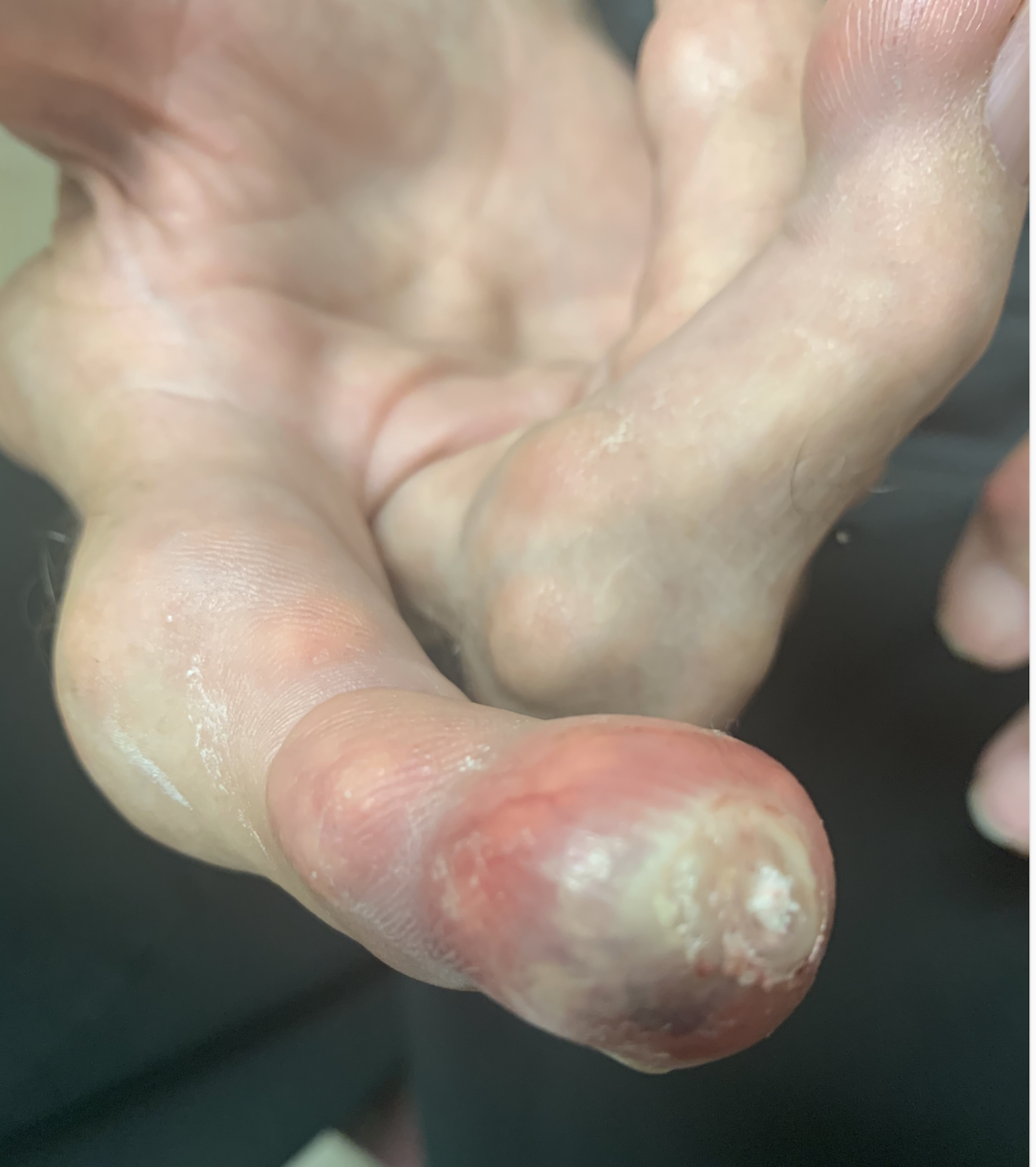

Supplement: Supplementary file 1 — Figure S1 [file CCR3-9-e05201-s007.png]

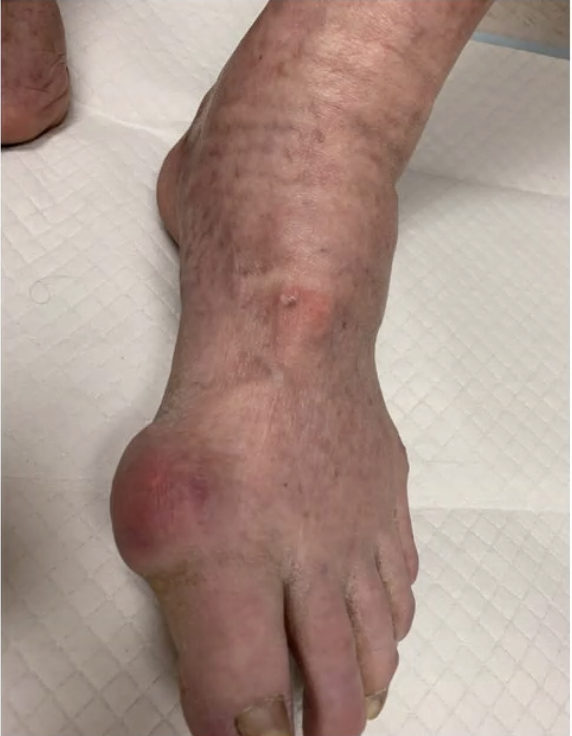

Supplement: Supplementary file 2 — Figure S2 [file CCR3-9-e05201-s006.png]

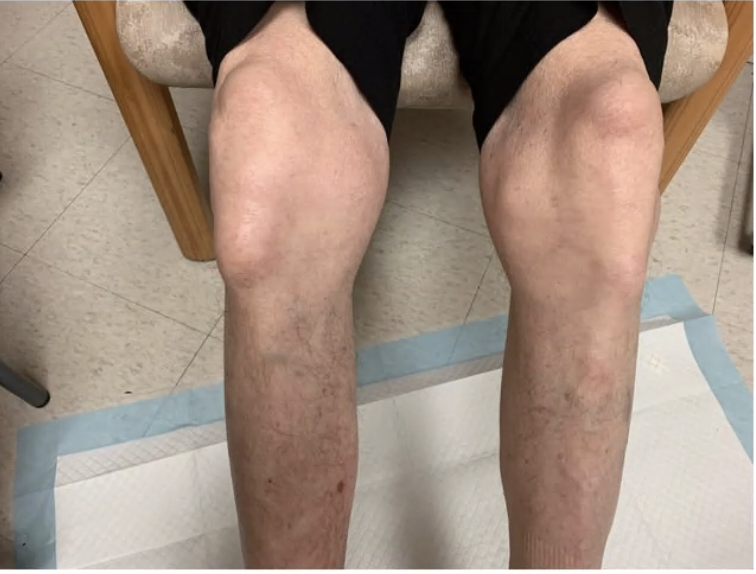

Supplement: Supplementary file 3 — Figure S3 [file CCR3-9-e05201-s005.png]

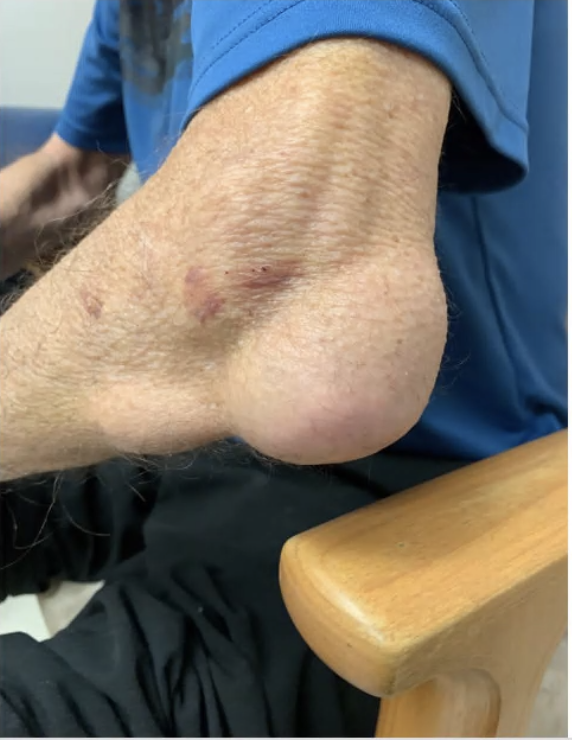

Supplement: Supplementary file 4 — Figure S4A [file CCR3-9-e05201-s003.png]

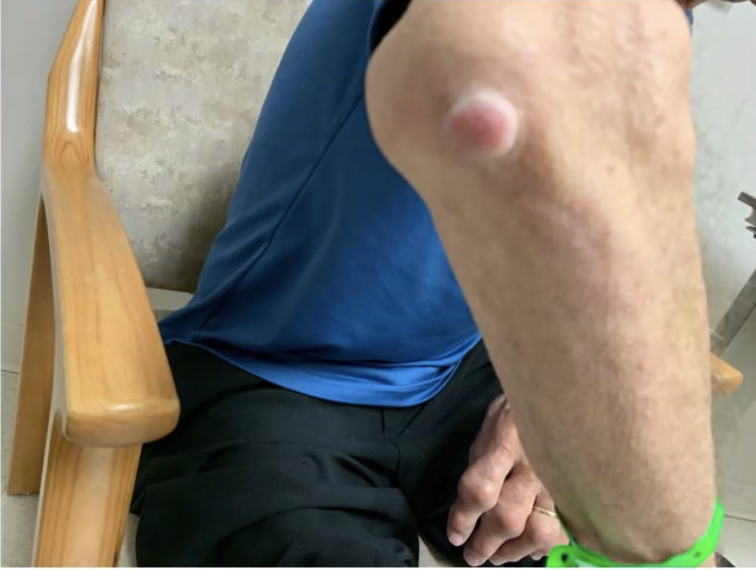

Supplement: Supplementary file 5 — Figure S4B [file CCR3-9-e05201-s001.png]

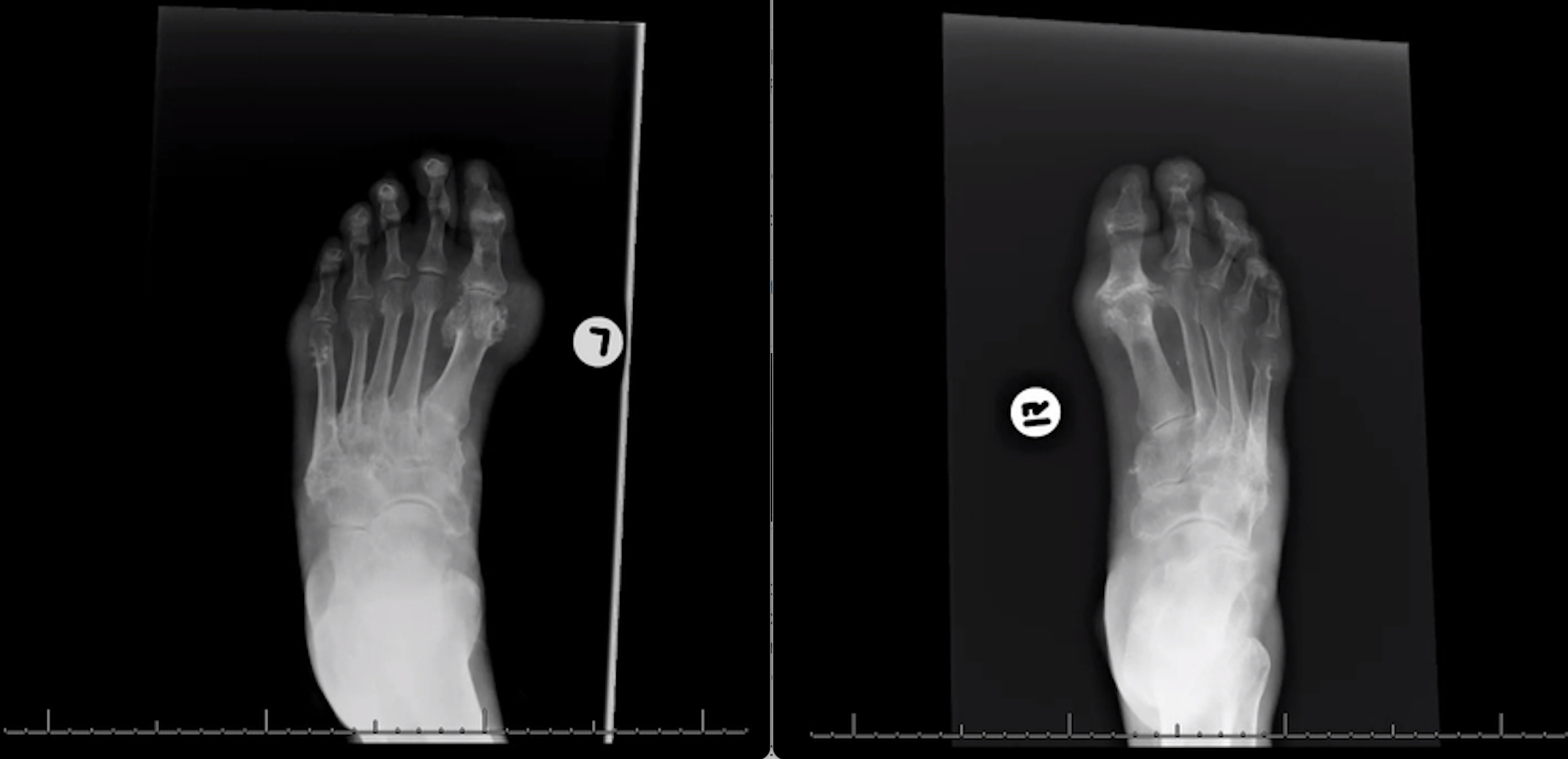

Supplement: Supplementary file 6 — Figure S5 [file CCR3-9-e05201-s004.png]

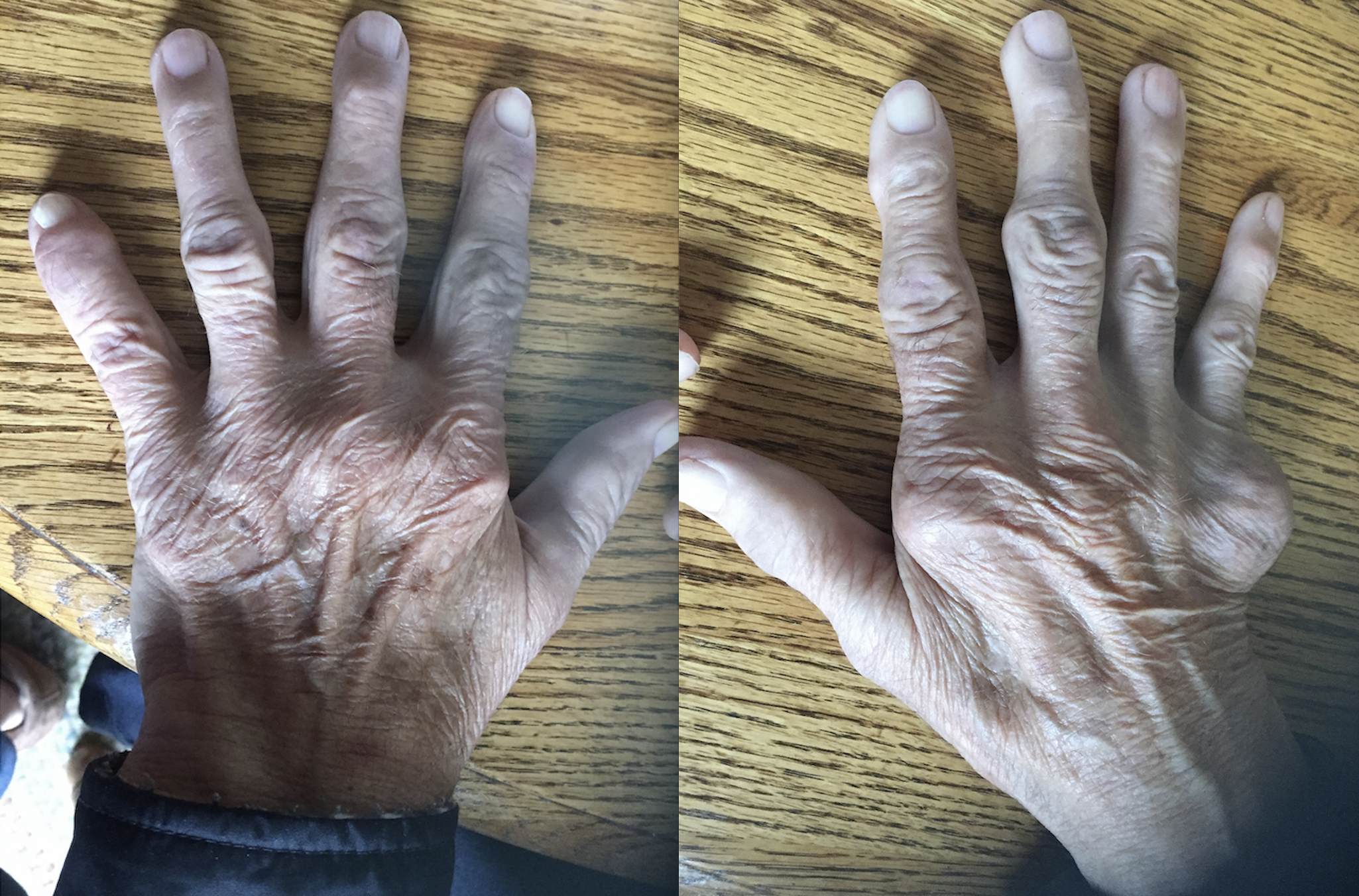

Supplement: Supplementary file 7 — Figure S6 [file CCR3-9-e05201-s002.png]
